# Coding RNA Sequencing of Equine Endometrium during Maternal Recognition of Pregnancy

**DOI:** 10.3390/genes10100749

**Published:** 2019-09-25

**Authors:** Kristin M. Klohonatz, Stephen J. Coleman, Alma D. Islas-Trejo, Juan F. Medrano, Ann M. Hess, Ted Kalbfleisch, Milton G. Thomas, Gerrit J. Bouma, Jason E. Bruemmer

**Affiliations:** 1 Department of Animal Sciences, Colorado State University, Fort Collins, CO 80523, USA; kmk5057@gmail.com (K.M.K.); stephen.coleman@colostate.edu (S.J.C.); Milt.Thomas@colostate.edu (M.G.T.); 2 Department of Animal Science, University of California Davis, Davis, CA 95616, USA; adislas@ucdavis.edu (A.D.I.-T.); jfmedrano@ucdavis.edu (J.F.M.); 3 Department of Statistics and Bioinformatics, Colorado State University, Fort Collins, CO 80523, USA; ann.hess@colostate.edu; 4 Department of Veterinary Science, Gluck Equine Research Center, University of Kentucky, Lexington, KY 40503, USA; ted.kalbfleisch@uky.edu; 5 Department of Biomedical Sciences, Animal Reproduction and Biotechnology Laboratory, Colorado State University, Fort Collins, CO 80523, USA; Gerrit.Bouma@ColoState.EDU; 6 Department of Animal Sciences, Colorado State University, 259 Animal Sciences, 1171 Campus Delivery, Fort Collins, CO 80523-1171, USA

**Keywords:** equine, transcriptome, pregnancy, maternal recognition of pregnancy

## Abstract

Equine maternal recognition of pregnancy (MRP) is a process whose signal remains unknown. During MRP the conceptus and endometrium communicate to attenuate prostaglandin F_2α_ (PGF) secretion, sparing the corpus luteum and maintaining progesterone production. Recognition of a mobile conceptus by the endometrium is critical by days 14–16 post-ovulation (PO), when endometrium produces PGF, initiating luteolysis. The objective of this study was to evaluate endometrial gene expression changes based upon pregnancy status via RNA sequencing. This experiment utilized a cross-over design with each mare serving as both a pregnant and non-mated control on days nine, 11, and 13 PO (*n* = 3/status/day). Mares were randomly assigned to collection day and pregnancy confirmed by terminal uterine lavage at the time of endometrial biopsy. Total RNA was isolated and libraries prepared using Illumina TruSeq RNA sample preparation kit. Reads were mapped and annotated using HISAT2 and Stringtie. Expression values were evaluated with DESEQ2 (*P* ≤ 0.05 indicated significance). On day nine, 11, and 13 there were 1435, 1435 and 916 significant transcripts, respectively. Multiple genes with splice variants had different expression patterns within the same day. These are the first data to evaluate the endometrial transcriptome during MRP on days nine, 11, and 13.

## 1. Introduction

Maternal recognition of pregnancy (MRP) in the horse is a complex process that involves communication between the conceptus and maternal endometrium. The equine conceptus does not attach to the endometrium until approximately day 35 post-ovulation (PO), so communication is occurring without attachment to prevent the endometrium from secreting prostaglandin F_2α_ (PGF_2α_), which causes luteal regression of the corpus luteum, ultimately eliminating the source of progesterone [1,2].

In both pregnant and non-pregnant mares, the hormonal profile stays the same until day 14 PO [3]. In the non-pregnant mare, oxytocin is released from both the posterior pituitary gland and endometrium. Oxytocin binds to endometrial receptors, which causes release of more oxytocin and subsequently, PGF_2α_ [4]. In pregnant mares, fertilization occurs in the oviduct, but the embryo does not enter the uterus until day six. At this time the embryo is surrounded by the zona pellucida. Following hatching, at approximately day seven, the embryo remains covered by an acellular, glycoprotein capsule [5]. This glycoprotein capsule remains intact until roughly day 16 when breakdown begins to occur [6]. Upon entering the uterus, the embryo is highly mobile, resulting from uterine contractions, reaching peak mobility between days 11–14 [5,7]. This mobility is necessary to delay secretion of PGF_2α_ [7,8,9]. By day 16 this mobility ceases and the embryo becomes fixed in a single location, but does not attach or invade [7]. Maternal recognition of pregnancy occurs between days 11–14 and is categorized as anti-luteolytic [7,10,11].

The signals for MRP in other species, such as Interferon tau and estradiol, have been tested in the horse, but do not have an impact on equine luteal function [12,13]. Unique to the horse, prostaglandin E2 is secreted by the conceptus in order for it to enter the uterus, but when infused into the uterus of non-pregnant mares, there is no effect [13]. Other experiments have infused coconut or peanut oil on day 10 of non-pregnant mares’ cycle and luteostasis was achieved [14]. This indicates that a component in the oil impacts the luteolytic pathway, but subsequent studies have failed to illicit the same response [15]. Research on MRP has evaluated transcriptional differences in the endometrium during and after MRP utilizing a microarray [16,17]. This research suggested that there were transcriptional differences occurring by day 14, but previous research has failed to robustly reveal candidates involved in MRP [16].

Equine maternal recognition of pregnancy is a multifaceted process that is still not well understood. All that is known is that the embryo must come into contact with over two-thirds of the endometrium to illicit the anti-luteolytic signal [8]. The objective of this study was to evaluate the endometrial transcriptome changes based upon pregnancy status before and during MRP.

## 2. Materials and Methods

### 2.1. Care and Management of Mares

Animal use was approved by the Colorado State University Institutional Animal Care and Use Committee. Mares (*n* = 9) were housed in group pens at Colorado State University Bud and Jo Adams Equine Reproduction Laboratory (Fort Collins, CO, USA). The mares were maintained on a dry lot and fed grass-alfalfa hay mix with free choice mineral and salt supplement. Mares were used in a paired, cross-over design in which each mare had a pregnant and non-pregnant (non-mated) cycle. Mares were monitored via transrectal palpation and ultrasonography to track follicular development every other day. To obtain samples from a pregnant mare, when a follicle reached 35 mm in diameter, or greater, the mare was inseminated with at least 500 × 10^6^ progressively motile sperm from stallions with proven fertility. Mares were monitored via transrectal ultrasonography every day and inseminated every other day until ovulation (day zero). For the non-mated cycle, the same procedure was followed with the exception of the insemination.

Mares were randomly assigned to collection day nine, 11, or 13 post-ovulation (PO) for both their pregnant (P+) and non-mated (NP) cycles. On the mares’ assigned day, each was evaluated via transrectal ultrasonography to confirm pregnancy status by visualization of an embryonic vesicle and terminal uterine lavage was completed. Endometrial samples were obtained non-surgically via a trans-cervical biopsy punch [18]. After embryo and/or biopsy collection, the mare received a luteolytic dose of PGF_2α_ (Estrumate, Merck Animal Health, Madison, NJ, USA, 250 mcg per dose). For the non-pregnant (non-mated) control cycle, the subsequent estrous cycle was utilized. After endometrial samples were obtained, each sample was rinsed in DPBS/Modified 1X (Hyclone Laboratories, Logan, UT, USA) and stored at −80 °C immediately.

### 2.2. RNA Isolation and Quantification

After collection, total RNA was isolated from all samples using TRI Reagent (Molecular Research Center, Cincinnati, OH, USA) for lysis and extraction and the RNeasy Mini Kit (Qiagen, Valencia, CA, USA) for purification. About 30 mg of frozen tissue was homogenized in TRI Reagent and incubated at room temperature for 10 min. Chloroform was added to the homogenate, vortexed, and incubated at room temperature for 8 min. The sample was centrifuged at 16,100× *g* for 15 min, which separated the sample into three distinct phases (RNA, DNA, and protein). The top aqueous RNA phase was transferred to a new 1.7 mL tube for isolation. RNA was isolated using the Qiagen RNeasy Mini Kit according to the manufacturer’s recommendations. All samples were treated with an RNase-Free DNase kit (Qiagen, Valencia, CA, USA) to remove DNA contamination. RNA purity and quantification were assessed using the NanoDrop Spectrophotometer ND-1000 (Thermo Scientific, Wilmington, DE, USA). Samples were used for analysis only if they had 260/280 and 260/230 values above 1.7 for RNA sequencing library preparation and PCR validation. 

### 2.3. RNA Sequencing

RNA-sequencing (cDNA) libraries were prepared using the Illumina TruSeq Sample Preparation Kit v2 (Illumina, San Diego, CA, USA) and 1 μg of total RNA from each sample following the manufacturer’s protocol. Briefly, adapters were ligated and samples were then reverse transcribed to form cDNA. Each sample was amplified with a specific barcoded PCR primer for sample identification purposes. Prepared libraries were sent to the University of California-Davis for quality control assessment and then to the University of California-Berkeley for sequencing. Single-end reads of 100 base-pairs were generated for each sample on an Illumina HiSeq 2000 (Illumina, San Diego, CA, USA). Sequences are available in the NCBI sequence read archive under BioProject PRJNA545717.

### 2.4. Bioinformatic Analysis

Bioinformatic analysis was performed on the Galaxy web platform [19] and used the public server at usegalaxy.org. Sequence quality was assessed by FastQC and results were aggregated and evaluated using MultiQC [20,21]. Trimmomatic was utilized to remove adapter sequence and low quality sequence [22]. Bases were removed if their quality score was below a threshold of 25. Reads were aligned to EquCab3.0 (NCBI accession GCF_002863825.1) using HISAT2 [23,24]. Stringtie was used for transcript assembly and quantification of both annotated and unannotated transcripts. Samples were analyzed individually and the results merged together and combined with the EquCab3 gene annotation (https://www.ncbi.nlm.nih.gov/genome/?term=txid9796[orgn]) to create a final transcript annotation file, which was then used for quantification of each sample [25]. Transcript read counts were analyzed within each day comparing samples from pregnant mares to non-pregnant mares utilizing DESeq2 within R [26]. To be considered for analysis, reads were present in at least two out of the three replicates in at least one of the two groups (P+ or NP). Data were normalized internally using DESeq2’s median of ratios method. The Benjamini Hochberg false discovery rate adjustment was used. Significance was assessed at *p* ≤ 0.05. 

Differentially expresses genes were evaluated within Ingenuity Pathway Analysis (IPA) to biological processes being targeted (QIAGEN Inc., Hilden, Germany), https://www.qiagenbioinformatics.com/products/ingenuity-pathway-analysis). Threshold values from DESeq2 were set at *p* ≤ 0.05 and fold change ≥ 1.5 in order to be utilized for IPA.

Table S1 contains the results of the analysis when reads were present in all three of the replicates in at least one of the two groups (P+ or NP).

## 3. Results

### 3.1. Sequencing Results

Quality filtering and removal of adapter sequence and low-quality sequence resulted in an average read length of 89 base pairs for all samples. Samples generated on average 33,810,516 reads (ranging from 29,450,126 to 39,054,008). Mapping efficiency to build three of the equine genome (EquCab3) was 94%.

### 3.2. Transcript Assembly and Analysis

**Figure 1:**
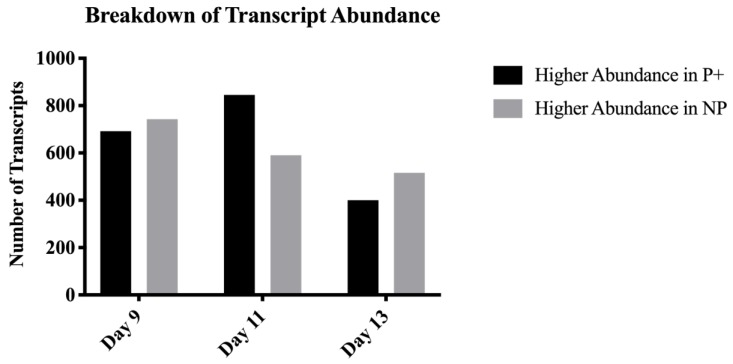
Transcript expression within each day from samples of endometrium from pregnant and non-pregnant mares. This figure demonstrates the breakdown of how many transcripts (*p* ≤ 0.05) were higher in abundance in samples from pregnant mares (P+) versus the numbers in samples from non-pregnant mares (NP).

**Figure 2:**
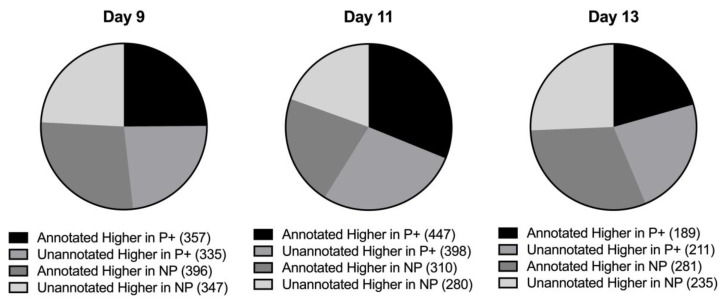
Breakdown of transcript abundance patterns for annotated and unannotated transcripts in equine endometrium from pregnant and non-pregnant mares. This figure illustrates out of all significant transcripts (*p* ≤ 0.05) identified each day, the number that were annotated or unannotated and in which pregnancy status (pregnant = P+, non-pregnant = NP) they were in higher abundance.

Available sequence generated from the endometrial samples from pregnant and non-pregnant mares at days nine, 11, and 13 identified 86,113, 82,449, and 81,787 transcripts, respectively. On day nine, there were a total of 1435 transcripts that differed (*p* ≤ 0.05) in abundance between endometrial samples from pregnant or non-pregnant mares, but 682 were unannotated. Of all significant transcripts, 693 were more abundant in samples from pregnant mares and 743 were more abundant in samples from non-pregnant mares (Figure 1). Seven hundred and fifty-three of the identified transcripts were previously annotated. Of these, 357 were more abundant in samples from pregnant mares and 396 were more abundant in samples from non-pregnant mares (Figure 2). 

On day 11, there were 1435 transcripts (639 genes; *p* ≤ 0.05) identified in endometrial samples. Of these transcripts, 845 were more highly abundant in samples from pregnant mares and 590 were more abundant in samples from non-pregnant mares (Figure 1). Of the 1435 transcripts, 678 were unannotated. On day 13, there were a total of 421 genes and 916 transcripts identified (*p* ≤ 0.05). Within these transcripts, 446 were unannotated. Of all of the significant transcripts identified, 400 were more highly abundant in samples from pregnant mares and 516 were more highly abundant in samples from non-pregnant mares. Figure 2 shows the breakdown of abundance between the annotated and unannotated significant transcripts. Table 1 displays the number of differentially expressed genes and transcripts identified on each day.

Many of the transcripts identified were not previously annotated in the equine genome. Of the differentially expressed transcripts, 682 (47.5%) identified on day nine, 678 (47.2%) identified on day 11, and 446 (48.7%) identified on day 13 were not previously annotated. Figure 2 shows the breakdown of abundance between the annotated and unannotated significant transcripts. Table 2 contains the top 20 significant transcripts for each day and Supplemental Tables S2–S4 describe all significant transcripts from days nine to 13, respectively.

### 3.3. Day 9

The most highly abundant non-ribosomal, gene in day nine endometrial samples from pregnant mares was Early Growth Response 1 (EGR1) and from endometrial samples from non-pregnant mares it was an unannotated transcript (MSTRG.8258.4) followed by annotated transcript (non-ribosomal), Homocysteine Inducible ER Protein with Ubiquitin Like Domain 1 (HERPUD1). Interestingly, the annotated transcript with the largest fold change (Log2 fold change = 13.77, more abundant in samples from pregnant mares) between samples from pregnant compared to non-pregnant mares was Hook Microtubule Tethering Protein 1 (HOOK1). Table 3 contains the top 10 annotated transcripts with the largest fold change between groups. 

**Figure 3:**
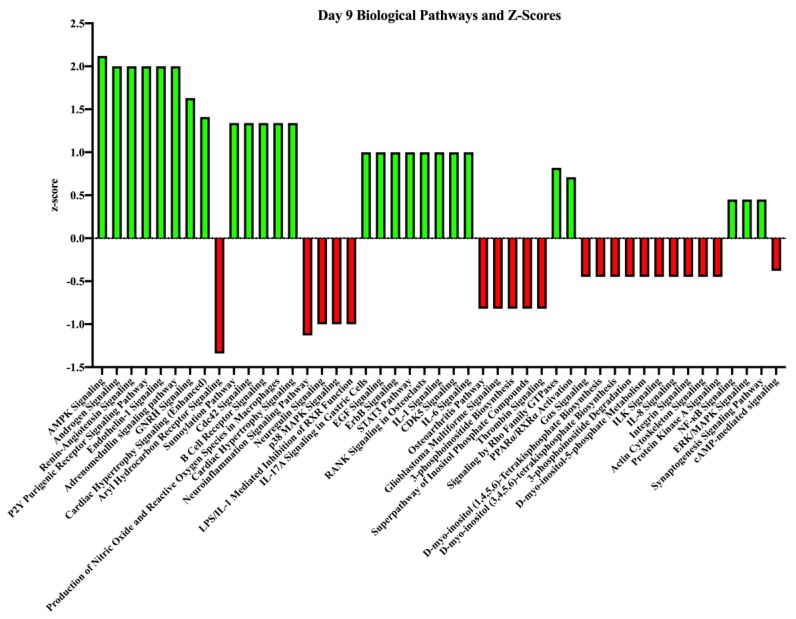
Top biological processes and z-scores on day nine in endometrial samples from pregnant and non-pregnant mares. These are the biological processes associated with the genes of higher abundance in the given pregnancy status on day nine. The z-score indicates the association strength with activating (green bars) or inhibiting (red bars) a pathway due to the sample being obtained from a pregnant mare.

Transcripts (*p* ≤ 0.05) were analyzed with IPA to determine the canonical pathways they have been associated with experimentally. Only annotated transcripts were used for this analysis. Some of the top biological pathways that were stimulated due to the presence of an embryo (P+) in our dataset on day nine were AMPK (AMP-activated protein kinase) signaling (z-score = 2.1), androgen signaling (z-score = 2.0) and GnRH signaling (z-score = 1.6). Some of the top biological pathways that were inhibited in our dataset on day nine included Aryl hydrocarbon receptor signaling (z-score = −1.3), neuroinflammation signaling (z-score = −1.1), and neuregulin signaling (z-score = −1.0). Other biological pathways of interest that were inhibited included ILK (integrin-linked kinase) signaling and actin cytoskeleton signaling. The biological processes with z-score associations are in Figure 3 and all associated biological processes are in Table S2.

### 3.4. Day 11

In samples from pregnant mares, the most abundant transcript was ATPase Na+/K+ Transporting Subunit Alpha 1 (ATP1A1) and the most abundant transcript in samples from non-pregnant mares was Aldehyde Dehydrogenase 1 Family Member A1 (ALDH1A1). The largest fold change (Log2 fold change = 27.5) difference between groups was unannotated transcript MSTRG.25465.6, more abundant in samples from pregnant mares. The largest fold change of an annotated transcript (Log2 fold change = 13.6) was Membrane Bound O-Acyltransferase Domain Containing 2 (MBOAT2), also more abundant in samples from pregnant mares. Interestingly, of the top ten transcripts with the largest fold change, only one was an annotated transcript, and only one was higher in abundance in samples from non-pregnant mares (unannotated transcript MSTRG.6103.11). The top ten fold changes of annotated transcripts are presented in Table 3.

**Figure 4:**
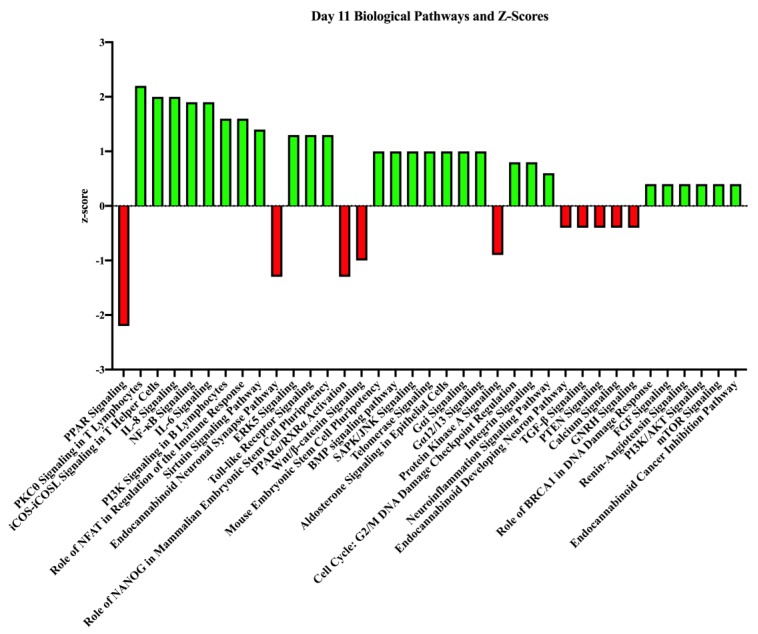
Top biological processes and z-scores on day 11 in endometrial samples from pregnant and non-pregnant mares. These are the biological processes associated with the genes of higher abundance in the given pregnancy status on day 11. The z-score indicates the association strength with activating (green bars) or inhibiting (red bars) a pathway due to the sample being obtained from a pregnant mare.

The biological pathways associated with all annotated transcripts (*p* ≤ 0.05) were evaluated. In total, transcripts on day 11 were associated with 395 biological processes, but only 82 were associated with z-scores indicating association with stimulating or inhibiting that biological process based upon pregnancy status (P+ or NP; Table S3). Figure 4 highlights some of those biological pathways. Some of the biological processes that were stimulated on day 11 due to pregnancy status (P+). These include protein kinase C theta (PKCθ) signaling in T lymphocytes, extracellular-signal-regulated kinase 5 (ERK5) signaling, and integrin signaling. 

The top biological pathway that was inhibited in this dataset on day 11 due to pregnancy status was peroxisome proliferator-activated receptor (PPAR) signaling (z-score = −2.2). Other biological processes that were inhibited included endocannabinoid neuronal synapse pathway, Wnt/β-catenin signaling, GnRH signaling, and TGF-β signaling. Some of the top biological pathways of interest are in Figure 4.

### 3.5. Day 13 

The most abundant transcript in samples from pregnant mares was an unannotated transcript (MSTRG.25465.3; expression value = 27,140 counts). This resulted in a Log2 fold change of 30.0 between samples from pregnant mares compared to non-pregnant mares. The most abundant transcript in samples from non-pregnant mares was Fos Proto-Oncogene, AP-1 Transcription Factor Subunit (FOS). The largest fold change was unannotated transcript MSTRG.25465.3. The largest fold change in annotated transcripts was Acyl-CoA Oxidase 1 (ACOX1; Log2 fold change = −14.1), more abundant in samples from non-pregnant mares. Interestingly, the second largest old change in annotated transcripts on day 13 was the same as on day 11, MBOAT2 (Log2 fold change = 13.9), more abundant in samples from pregnant mares. Table 3 contains the top ten fold changes for annotated transcripts.

**Figure 5:**
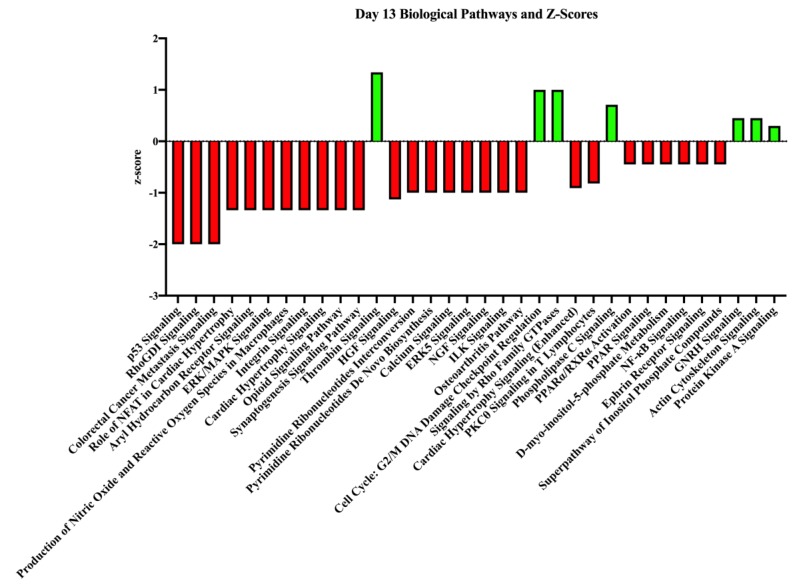
Top biological processes and z-scores on day 13 in endometrial samples from pregnant and non-pregnant mares. These are the biological processes associated with the genes of higher abundance in the given pregnancy status on day 13. The z-score indicates the association strength with activating (green bars) or inhibiting (red bars) a pathway due to the sample being obtained from a pregnant mare.

Interestingly, on day 13 only seven biological pathways were stimulated based upon pregnancy status in our dataset. These biological pathways included thrombin signaling, signaling by Rho family GTPases, GnRH signaling, and actin cytoskeleton signaling. In contrast, the most heavily inhibited biological process from our dataset on day 13 was p53 signaling (z-score = −2.0). Other inhibited biological processes of interest included RhoGDI signaling, ILK signaling, integrin signaling, and (in contrast to day 11) PPAR signaling. Figure 5 contains other biological pathways identified with these transcripts and the full list can be found in Supplemental Table S4.

### 3.6. Significant Transcripts on Days 9, 11 and 13

Interestingly there were only two transcripts that were significant on all three days (*p* ≤ 0.05). One transcript was unannotated, MSTRG.27075.4. It was more abundant across all three days in samples from pregnant mares (Log2 fold change = 4.2, 3.0 and 3.7, respectively). The other transcript was gene Gametogenetin Binding Protein 2 (GGNBP2). This gene was actually more abundant in samples from pregnant mares on days nine and 11 (Log2 fold change = 2.8 and 2.7, respectively) and more abundant in samples from non-pregnant mares on day 13 (Log2 fold change = −2.3).

## 4. Discussion

These are the first data reported that evaluated the transcriptome in equine endometrium across the duration of maternal recognition of pregnancy (MRP). This study utilized days nine, 11, and 13 in order to evaluate the transcriptome before, during and after MRP. What is also unique about this study is that it not only evaluated the genes, but also the transcripts (splice variants) within those genes. In addition to identifying known genes, we also identified a population of unannotated genes/transcripts.

An interesting phenomenon noted during this analysis was the change in expression status between variants of a gene. Multiple genes had many transcripts that were significant within a day, but sometimes the transcripts would vary in whether they were more highly expressed in samples from pregnant mares or non-pregnant mares. An example was USP36 on day nine, ubiquitin specific peptidase 36. Transcript 1 was more abundant in samples from pregnant mares whereas transcript 2 was more abundant in samples from non-pregnant mares. This occurrence of transcripts of the same gene having opposing expression patterns happened with many genes throughout the results suggesting that alternative splicing could be occurring in the endometrium during this time frame. Alternative splicing is the combination of different splice sites joining together to form the gene [27]. Others have shown that unbalanced splice variants can result in tumors within tissues [28]. Introns are removed by a protein known as a spliceosome [29]. Spliceosomes are an assembly of five small nuclear ribonucleoproteins (snRNP). These snRNP are referred to as U1, U2, U4, U5, and U6 small nuclear RNA (snRNA) [29]. More research is needed to understand the significance of this alternative splicing and determine if snRNA are present in the endometrium, resulting in alternative splicing.

Another observation from this study was the large number of genes/transcripts that were present in only samples from pregnant or non-pregnant mares. During this time frame, pregnant and non-pregnant mares have the same hormonal profile [3]. Therefore, this gene expression in one sample versus the other has to occur due to the presence of an embryo, indicating an active role in maternal recognition of pregnancy. Further research of these genes is required to understand the significance of the genes being exclusively expressed in one sample.

A biological pathway that was targeted by many transcripts, on multiple days, was integrin signaling. Subsequently, it was also a pathway of great interest in regard to maternal recognition of pregnancy. Integrin linked kinase (ILK) signaling was also heavily impacted by transcripts within our study. Previous reports suggested that focal adhesions are present in equine endometrium during the time of maternal recognition of pregnancy and serum samples from pregnant and non-pregnant mares contain exosomes that are transporting microRNA (miRNA) targeting focal adhesions [30,31]. Focal adhesions are integrin receptors on the plasma membrane that sense and transduce mechanical forces from the extracellular matrix to a biochemical signal within the cell [32,33]. The two main kinases responsible for these biochemical signals within the cell are focal adhesion kinase (FAK) and integrin linked kinase (ILK) [33,34].

In this study, integrin signaling was inhibited on days nine and 13 and enhanced on day 11. The reason for this variability is due to the genes contributing to the biological process on the specified days. On day nine one of the key contributors to inhibition of integrin signaling is RHOH. This gene is required to maintain integrins, specifically integrin LFA-1 [35]. In our dataset, RHOH is not present in samples from pregnant mares. Without this protein present, focal adhesions may not be able to be maintained, ultimately decreasing integrin signaling. On day 11, integrin signaling is stimulated due to pregnancy status (P+). Multiple genes are more abundant in samples from pregnant mares, contributing to increased integrin signaling. Two of these genes are GRB2 associated binding protein 1 (GRB1) and Rho guanine nucleotide exchange factor 7 (ARHGEF7). These two genes are associated with organization and formation of focal adhesions [36,37]. Integrin Beta 6 (ITGB6) is an integrin located in the cellular membrane that is associated with focal adhesions. Previously, it has been identified at the interface between the porcine conceptus and trophoblast [38]. ABL proto-oncogene 1, non-receptor tyrosine kinase (ABL1) is triggered by activation of integrins such as ITGB6 [39]. Other integrins that may activate ABL1 that are present in our dataset in higher abundance in samples from pregnant mares in Integrin Alpha 10 (ITGA10). Previous studies in our lab have also noted a higher abundance of ITGA10 in endometrial samples from pregnant mares [30]. A gene was identified that also interacts directly with FAK, phosphatidylinositol-4,5-biphosphate 3-kinase catalytic subunit delta (PIK3CD) [40,41]. Most of these genes were determined to be more abundantly expressed (*p* ≤ 0.05) in samples from pregnant mares on day 11. On day 13 the main gene responsible for this pathway enrichment was RAPGEF, rap guanine nucleotide exchange factor 1. This gene is more closely related to actin dynamics within the cell and adheres to focal adhesions versus signaling from focal adhesions [42]. This could be the reason for the decrease in integrin signaling.

Although not significant in this study between groups, another interesting observation was the identification of CATSPERD, CATSPERG, and CATSPERB, all subunits of CATSPER that are required to form a functional ion channel, within the endometrium [43]. Previously, CATSPER, a sperm calcium transporter, was thought to be unique to the sperm [44]. To our knowledge, this is the first report to CATSPER outside of sperm, and more specifically within the endometrium.

## 5. Conclusions

This is the first study evaluating equine endometrium before, during, and after maternal recognition of pregnancy utilizing RNA sequencing. A large number of genes/transcripts were identified that were unique to pregnancy status and day, including many novel transcripts with unknown functions. Interestingly, alternative splicing was identified, yet the importance of these needs to be determined. Further research is needed to determine the role of the genes identified in this study in order to elucidate the signaling that is occurring during maternal recognition of pregnancy.

## Figures and Tables

**Table 1:** Significant genes and transcripts within days nine, 11, and 13 of equine endometrium from pregnant and non-pregnant mares. This table breaks down the number of significant (*p* ≤ 0.05) genes and transcripts identified within each day. It also states out of all the transcripts identified how many were previously unannotated.

	Genes	Transcripts	Unannotated Transcripts
Day 9	634	1435	682
Day 11	639	1435	678
Day 13	421	916	446

**Table 2:** Top 20 transcripts ranked by significance for each day in equine endometrium among samples from pregnant and non-pregnant mares.

Day 9				Day 11				Day 13			
All		Annotated		All		Annotated		All		Annotated	
Transcript	*p*-Value	Transcript	*p*-Value	Transcript	*p*-Value	Transcript	*p*-Value	Transcript	*p*-Value	Transcript	*p*-Value
ZFHX3	4.00 × 10^−76^	ZFHX3	4.00 × 10^−76^	ACTN4	6.03 × 10^−55^	ACTN4	6.03 × 10^−55^	**MSTRG.15333.5**	3.09 × 10^−36^	ACOX1	7.14 × 10^−24^
ERBB2	2.49 × 10^−56^	ERBB2	2.49 × 10^−56^	**MSTRG.3211.2**	6.90 × 10^−45^	**AGRN**	2.46 × 10^−40^	ACOX1	7.14 × 10^−24^	SORBS3	3.23 × 10^−17^
**NF1**	2.49 × 10^−56^	**NF1**	2.49 × 10^−56^	**MSTRG.6009.2**	8.48 × 10^−42^	C16H3orf67	1.43 × 10^−33^	SORBS3	3.23 × 10^−17^	SERPINB9	2.70 × 10^−15^
**MSTRG.27804.1**	9.13 × 10^−53^	MKLN1	3.03 × 10^−39^	**AGRN**	2.46 × 10^−40^	BTF3L4	5.84 × 10^−30^	**MSTRG.22183.4**	6.65 × 10^−17^	**MTMR2**	7.01 × 10^−15^
**MSTRG.15604.11**	1.57 × 10^−50^	**LACTB**	1.84 × 10^−37^	**MSTRG.1680.8**	6.10 × 10^−35^	**PITPNA**	6.19 × 10^−27^	**MSTRG.17471.3**	6.54 × 10^−16^	DOCK1	1.32 × 10^−14^
MKLN1	3.03 × 10^−39^	**EGR1**	8.44 × 10^−27^	MSTRG.26234.10	2.27 × 10^−34^	**BICRAL**	2.66 × 10^−23^	**MSTRG.28537.13**	1.28 × 10^−15^	**FAM20B**	7.12 × 10^−14^
**MSTRG.13994.1**	1.56 × 10^−38^	AKAP11	2.57 × 10^−24^	C16H3orf67	1.43 × 10^−33^	**STX3**	3.11 × 10^−20^	MSTRG.20351.7	1.64 × 10^−15^	TTC28	1.59 × 10^−13^
**LACTB**	1.84 × 10^−37^	**AKAP11**	1.40 × 10^−23^	BTF3L4	5.84 × 10^−30^	**PATZ1**	2.69 × 10^−19^	SERPINB9	2.70 × 10^−15^	HIP1	1.77 × 10^−13^
**MSTRG.19114.13**	6.66 × 10^−32^	COMMD4	3.12 × 10^−23^	**PITPNA**	6.19 × 10^−27^	ZNF605	4.10 × 10^−19^	MSTRG.14125.20	7.01 × 10^−15^	USP42	1.03 × 10^−12^
MSTRG.13516.12	2.06 × 10^−30^	**NXT2**	5.60 × 10^−23^	MSTRG.5600.1	1.31 × 10^−26^	**FAM104A**	1.44 × 10^−18^	**MTMR2**	7.01 × 10^−15^	**UNK**	1.18 × 10^−12^
**EGR1**	8.44 × 10^−27^	**R3HDM2**	8.69 × 10^−22^	**BICRAL**	2.66 × 10^−23^	**TNPO1**	2.22 × 10^−17^	DOCK1	1.32 × 10^−14^	**TLDC1**	1.84 × 10^−11^
MSTRG.14475.5	4.73 × 10^−26^	**NCBP1**	5.71 × 10^−20^	**MSTRG.25435.2**	9.22 × 10^−21^	**MME**	8.32 × 10^−17^	**MSTRG.5354.16**	2.93 × 10^−14^	**ZBTB37**	2.32 × 10^−11^
**MSTRG.10550.2**	2.08 × 10^−25^	**C1H1orf198**	5.81 × 10^−20^	**STX3**	3.11 × 10^−20^	**TMED8**	3.81 × 10^−16^	**MSTRG.15333.7**	6.54 × 10^−14^	C7H11orf54	4.06 × 10^−11^
AKAP11	2.57 × 10^−24^	NHS	6.50 × 10^−20^	**MSTRG.2169.2**	3.76 × 10^−20^	**RABGAP1**	7.47 × 10^−16^	**FAM20B**	7.12 × 10^−14^	KDM7A	9.32 × 10^−11^
**MSTRG.10469.4**	2.57 × 10^−24^	**PHF20**	2.45 × 10^−19^	**PATZ1**	2.69 × 10^−19^	PLA2G2C	1.33 × 10^−15^	**MSTRG.17032.9**	9.35 × 10^−14^	ATRX	9.32 × 10^−11^
**AKAP11**	1.40 × 10^−23^	C13H7orf26	7.43 × 10^−19^	ZNF605	4.10 × 10^−19^	LOC100064842	2.16 × 10^−15^	TTC28	1.59 × 10^−13^	LARS	2.45 × 10^−10^
**MSTRG.19003.10**	1.67 × 10^−23^	**RPSA**	1.26 × 10^−18^	**FAM104A**	1.44 × 10^−18^	DCAF6	2.73 × 10^−15^	HIP1	1.77 × 10^−13^	PHRF1	3.70 × 10^−10^
COMMD4	3.12 × 10^−23^	**PIGM**	1.60 × 10^−18^	**MSTRG.25230.44**	4.50 × 10^−18^	FBXO31	3.97 × 10^−15^	**MSTRG.26578.1**	6.78 × 10^−13^	**ATP11C**	7.35 × 10^−10^
**MSTRG.19003.12**	4.22 × 10^−23^	TEP1	3.64 × 10^−18^	MSTRG.22573.1	7.13 × 10^−18^	**MYO1C**	7.40 × 10^−15^	USP42	1.03 × 10^−12^	SLC25A25	1.18 × 10^−9^
MSTRG.13065.7	5.14 × 10^−23^	**SSH2**	1.22 × 10^−17^	**MSTRG.25564.2**	1.99 × 10^−17^	**NCOA2**	1.25 × 10^−14^	**MSTRG.3924.5**	1.18 × 10^−12^	CCDC181	1.23 × 10^−9^

This table shows the top 20 most significantly differentially expressed transcripts within each day. The table includes two groups. The first containing all transcripts for that day and the second containing only annotated transcripts. A transcript in bold indicates that it was more highly abundant in samples from pregnant mares.

**Table 3:** Top 10 annotated transcript fold changes for days nine, 11, and 13 in equine endometrium.

Day 9			Day 11			Day 13		
Transcript	Log2 FC	*p*-Value	Transcript	Log2 FC	*p*-Value	Transcript	Log2 FC	*p*-Value
HOOK1	13.8	2.76 × 10^−2^	MBOAT2	13.6	2.93 × 10^−2^	ACOX1	−14.1	7.14 × 10^−24^
GOLGB1	−13.8	2.79 × 10^−2^	NF1	-13.5	3.26 × 10^−2^	MBOAT2	13.9	3.42 × 10^−2^
AKAP11	13.1	1.40 × 10^−23^	PTPN4	13.5	4.45 × 10^−5^	YWHAZ	13.3	4.71 × 10^−2^
USF3	−12.5	9.02 × 10^−10^	PRKAA2	-13.3	3.72 × 10^−2^	ZBTB37	12.2	2.32 × 10^−11^
PFKFB3	11.7	2.22 × 10^−10^	PHC3	13.0	4.64 × 10^−2^	TTC28	−11.9	1.59 × 10^−13^
PIGM	11.7	1.60 × 10^−18^	TRANK1	13.0	4.68 × 10^−2^	MTMR2	11.9	7.01 × 10^−15^
SSH2	11.6	1.22 × 10^−17^	NFAT5	12.6	9.18 × 10^−10^	ULK2	−11.8	7.92 × 10^−4^
FUK	11.5	7.51 × 10^−15^	STX3	12.5	3.11 × 10^−20^	MSI2	−11.7	1.41 × 10^−5^
TTBK2	11.5	2.00 × 10^−3^	TLL1	12.4	3.35 × 10^−13^	KANK2	11.6	1.38 × 10^−6^
PPP6R2	11.1	7.51 × 10^−15^	TMEM181	12.1	1.57 × 10^−9^	NACC2	11.3	3.92 × 10^−3^

This table demonstrates the top ten annotated transcripts for each day ranked based upon the fold change (FC). A positive fold change indicates higher abundance in samples from pregnant mares and a negative fold change indicates higher abundance in samples from non-pregnant mares.

## Supplementary Materials

The following are available online at https://www.mdpi.com/2073-4425/10/10/749/s1, Table S1: All significant transcripts for analysis performed when reads were present in all three replicates in one of the two groups (P+ or NP) on days 9, 11 and 13, Table S2: All significant transcripts (*P* ≤ 0.05; annotated and unannotated transcripts) and biological pathways (for annotated transcripts) in equine endometrium from pregnant and non-pregnant mares on day 9 post-ovulation., Table S3: All significant transcripts (*P* ≤ 0.05; annotated and unannotated transcripts) and biological pathways (for annotated transcripts) in equine endometrium from pregnant and non-pregnant mares on day 11 post-ovulation. Table S4: All significant transcripts (*P* ≤ 0.05; annotated and unannotated transcripts) and biological pathways (for annotated transcripts.
